# Missense variants in *CYP4B1* associated with increased risk of lung cancer among Chinese Han population

**DOI:** 10.1186/s12957-023-03223-2

**Published:** 2023-11-11

**Authors:** Yongqin Yang, Shan Yuan, Shouchun Yan, Kuaini Dong, Yonghui Yang

**Affiliations:** 1 Department of General Surgery, Xi’an Yanliang 630 Hospital, Shaan Xi, China; 2 Department of Laboratory, Xi’an Yanliang 630 Hospital, East Renmin Road, Yanliang District, Xi’an City, 710000 Shaanxi Province China; 3 Department of Emergency Medicine, The Second Affiliated Hospital of Shaanxi University of Chinese Medicine, Shaan Xi, China

**Keywords:** Lung cancer, *CYP4B1*, Missense variants, Chinese Han population, Susceptibility

## Abstract

**Introduction:**

Understanding the etiology and risk factors of lung cancer (LC) is the key to developing scientific and effective prevention and control strategies for LC. *CYP4B1* genetic polymorphism has been reported to be associated with susceptibility to various diseases. We aimed to explore the association between *CYP4B1* genetic variants and LC susceptibility.

**Methods:**

One thousand three hundred thirty-nine participants were recruited to perform an association analysis through SNPStats online software. Statistical analysis of this study was mainly completed by SPSS 22.0 software. False-positive report probability analysis (FPRP) to detect whether the positive findings were noteworthy. Finally, the interaction of SNP-SNP in LC risk was evaluated by multi-factor dimensionality reduction.

**Results:**

We found evidence that missense variants in *CYP4B1* (rs2297810, rs4646491, and rs2297809) are associated with LC susceptibility. In particular, genotype GA of *CYP4B1*-rs2297810 was significantly associated with an increased risk of LC in both overall and stratified analyses (genotype GA: OR (95% CI) = 1.35 (1.08-1.69), *p* = 0.010). *CYP4B1*-rs4646491 (overdominant: OR (95% CI) = 1.30 (1.04-1.62), *p* = 0.023) and *CYP4B1*-rs2297809 (genotype CT: OR (95% CI) = 1.26 (1.01-1.59), *p* = 0.046) are also associated with an increased risk of LC. FPRP analysis showed that all positive results in this study are noteworthy findings

**Conclusion:**

Three missense variants in *CYP4B1* (rs2297810, rs4646491, and rs2297809) are associated with increasing risk of LC.

**Supplementary Information:**

The online version contains supplementary material available at 10.1186/s12957-023-03223-2.

## Introduction

A systematic analysis of the Global Burden of Disease (GBD) study (2019) showed that the global burden of cancer is substantial and growing [1]. A study comparing the latest cancer profiles in China and the United States found that the most common cancer is lung cancer in China and breast cancer in the United States, with lung cancer being the leading cause of cancer deaths in both countries [2]. According to World Health Organization statistics, the lung cancer worldwide average annual mortality rate is very high, it has brought serious adverse to human health and the social economy [3]. Therefore, the urgency of lung cancer prevention and control should be widely recognized and paid great attention to. Understanding the etiology and risk factors of lung cancer is the key to developing scientific and effective prevention and control strategies for lung cancer.

Tobacco is considered to be one of the main reasons affecting the occurrence and development of lung cancer [4]. However, it is not the only reason. Many studies have found that the lung cancer risk of females among Chinese with low smoking rates is about the same as that of females with high smoking rates in Western European countries [5, 6]. Therefore, in addition to smoking, there are other factors that may affect the occurrence and development of lung cancer, such as individual genetic factors and lampblack [7–9]. Researchers around the world have been studying the risk factors for lung cancer and ways to prevent it. Although there is no effective method to completely prevent lung cancer at present, it is recognized to reduce the risk of lung cancer by reducing exposure to risk factors and implementing precise and personalized prevention strategies. Therefore, it is particularly urgent to find new biomarkers and more accurate predictors of lung cancer to provide better diagnosis and prognosis for lung cancer patients [10].

SNP (single nucleotide polymorphism) is the most common single nucleotide variation among individuals and occurs in more than 1% of individual genomes in a population. Among the variations in the human genome, more than 90% belong to this kind of variation [11, 12]. As third-generation genetic markers, SNPs have the advantages of high density, representativeness, stability, etc. Therefore, they have been used in genetic analysis to locate quantitative trait loci (QTL) in lung cancer and other diseases, and a large number of related single nucleotide polymorphism loci have been identified [13, 14]. A large number of studies have reported that SNPs can change or regulate the function of genes, and play an important role in diseases including lung cancer. Such as, SNP rs35705950 is associated with familial interstitial pneumonia and idiopathic pulmonary fibrosis, and is involved in the pathogenesis of pulmonary fibrosis by regulating the expression of MUC5B in the lung [15]; SNP rs769236 may be involved in the occurrence of colon cancer, liver cancer and lung cancer by influencing the regulation of CCNA2 expression, and this SNP has been shown to be a valuable biomarker for evaluating individual patients' susceptibility to cancer [16]. SNP rs12587742 is involved in the occurrence of lung cancer by up-regulating the mRNA expression of DCAF4 and reducing its methylation status [17]. Although many genetic polymorphisms associated with lung cancer susceptibility have been identified, the molecular mechanisms involved in the development and development of lung cancer are still unclear. Screening SNPS related to the development of lung cancer in specific populations will lay a theoretical foundation for studying the molecular mechanism of lung cancer. And then further promote the individualized prevention and treatment of lung cancer.

Cytochrome P450 (CYP) family is a typical phase I drug metabolizing enzyme located in the inner mitochondrial membrane or the endoplasmic membrane of eukaryotic cells [18]. A variety of CYP proteins encoded by the human genome are responsible for the metabolism of many endogenous and exogenous compounds [19–21]. Several previous studies have reported the potential of CYP polymorphisms in cancer treatment. CYPs gene polymorphisms such as CYP1A1, CYP1B1, CYP2E1, CYP2D6, and CYP3A4 have been reported may be play an important role in chemotherapy and survival in lung cancer patients [22]. In the CYP family, CYP4 enzymes are involved in the metabolism of fatty acids, which is associated with susceptibility to genetic diseases [18]. A recent study using the Cancer Genome Atlas (TCGA) project and gene Expression Synthesis (GEO) database showed that *CYP4B1* is a potential therapeutic target for lung adenocarcinoma [23]. Fat is an important source of energy during tumorigenesis [24]. Studies have found that reprogramming of fatty acid metabolism plays an important role in the development of several cancers, including lung cancer [25]. Metabolic reprogramming is one of the hallmarks of tumor cells [26]. It is of great significance to understand the mechanism of metabolic reprogramming, which determines how we target metabolic reprogramming to treat cancer. The association between *CYP4B1* genetic polymorphism and susceptibility to prostate cancer, bladder cancer or other cancers has been reported [18, 27, 28]. However, no studies have reported the association between *CYP4B1* genetic polymorphism and lung cancer risk in the Chinese Han population. In conclusion, our study aimed to investigate the association between *CYP4B1* single nucleotide polymorphism (SNP) and susceptibility to lung cancer. We also conducted stratified analysis according to the potential risk factors related to the development of lung cancer, such as age, sex, smoking/drinking, tumor staging, tumor metastasis, tumor type, and so on, so as the relationship between *CYP4B1* SNP and these potential risk factors will be evaluated. This study will provide a data supplement for exploring novel biomarkers associated with susceptibility to lung cancer. Due to the potential role of *CYP4B1* in fatty acid metabolism, the study will help to explore the molecular mechanism of the occurrence and development of lung cancer and then lay a theoretical foundation for targeted metabolic reprogramming to treat lung cancer.

## Materials and methods

### Sample source

We recruited 1339 participants, including 668 lung cancer patients and 671 healthy individuals, for a case-control study. The 1339 participants were all recruited in the same hospital (Xi’an Yanliang 630 Hospital). We continuously recruited 668 LC patients in the outpatient or inpatient department of Respiratory and Critical Care Medicine. The case group consisted of patients diagnosed with lung cancer by pathological examination. Exclusion criteria included: (1) have a history of other cancers; (2) metastatic cancers from different cancers; (3) Secondary cases of lung cancer, or recurrent cases. During the same period, 671 healthy individuals were recruited in the health examination center as the control group in this study. All subjects were unrelated to Han Chinese. A total of 10 ml venous blood was collected from each subject using a vacuum blood collection tube containing EDTA-K2 anticoagulant (Solarbio, Beijing, China: YA1293). And they did not receive radiometabolic therapy prior to blood collection. Demographic information of all patients was obtained by reviewing medical records, and environmental exposure factors such as smoking were obtained by face-to-face questionnaire survey. The demographic data of the control group were obtained from the records of the physical examination center, and environmental exposure factors such as smoking were obtained through a face-to-face questionnaire survey. The contents of epidemiological survey data are as follows: name, sex, age, smoking status, etc. This study has been approved by the ethics committee of Xi’an Yanliang 630 Hospital before the beginning.

### Selection of SNPs

**Fig. 1:**
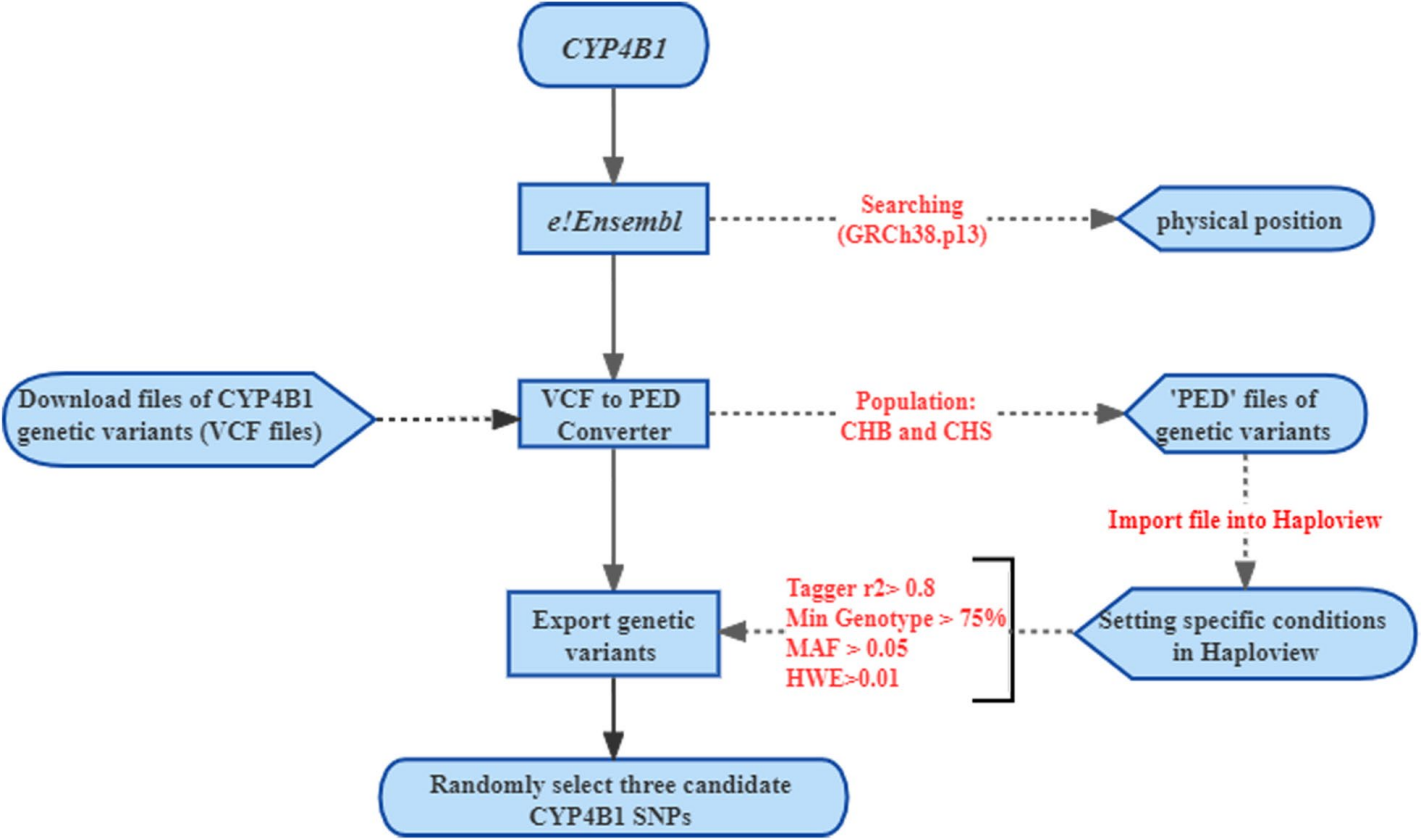
Flow chart for screening candidate SNPs

The selection process is shown in Fig. 1. First, we used the online tool to obtain the physical position of the *CYP4B1* on the Chromosome 1: 46,757,838-46,819,413 (e!GRCh38.p13: http://asia.ensembl.org/Homo_sapiens/Info/Index). Then, we chose CHB and CHS population and used the online converter window (VCF to PED: http://grch37.ensembl.org/Homo_sapiens/Tools/VcftoPed) to download the related files of *CYP4B1*genetic variants. Finally, after setting specific conditions (Tagger r^2^ > 0.8, Min Genotype > 75%, MAF> 0.05, and HWE> 0.01), three candidate *CYP4B1* SNPs were selected over Haploview software (rs2297810, rs4646491, and rs2297809).

### DNA extraction and genotyping

In this study, we chose the kit (GoldMag Co. Ltd. Xi’an, China) to complete the extraction and purification of genomic DNA, and the specific experimental steps were carried out according to the instructions. According to the DNA sequence of *CYP4B1*, all primers (amplification primers or extension primers) for candidate genetic loci can be designed by MassARRAY Assay Design software (All primers can be seen in Supplemental Table 1). Genotyping was completed by the MassARRAY ® -IPLEX SNP genotyping technology [29].

5% DNA samples were randomly selected for repeated experiments. The repetition rate of the results should reach >99% to ensure the reliability and repeatability of the experimental results.

### Prediction of association between genotype and gene expression level

In this study, the UALCAN online database (http://ualcan.path.uab.edu/analysis.html) [30] was used to analyze the expression differences of *CYP4B1* between normal lung tissues and LC tissues. We also analyzed the effect of *CYP4B1* gene expression on the prognosis of LC by the OncoLnc database (http://www.oncolnc.org/) [31]. Finally, the Genotype-Tissue Express (GTEx) database [32] was used to predict the association between genotypes of candidate genetic loci. and expression level of *CYP4B1* (https://gtexportal.org/home/).

### Data analysis

We used online software to predict the potential function of candidate SNPs (HaploReg v4.1: https://pubs.broadinstitute.org/mammals/haploreg/haploreg.php). And we obtained the information of candidate SNPs through dbSNP online database (https://www.ncbi.nlm.nih.gov/snp/). Statistical analysis of this study was mainly completed by SPSS 22.0 software (SPSS Inc., Chicago, IL, USA). In this study, the association between LC risk and candidate genetic polymorphisms was completed by SNPStats (https://www.snpstats.net/start.htm?q=snpstats/start.htm). We mainly evaluated the impact of candidate SNPs on the risk of LC over odds ratios (OR) and 95% confidence intervals (CI). We used the ‘Forest Mapping Tool’ in Sangerbox 3.0 online software to draw the forest map, and showed the positive results found in the hierarchical analysis on the map (http://sangerbox.com/home.html). In order to avoid the influence of confounding factors on the results, all the results were adjusted by confounding factors, such as age, gender, smoking, or drinking. In addition, we also used false-positive report probability (FPRP) analysis to detect whether all positive results are noteworthy at a prior probability level of 0.25 and an FPRP threshold of 0.2 [33]. Finally, the interaction of candidate SNPs in LC risk was evaluated by multi-factor dimensionality reduction (MDR). The *p* < 0.05 indicated statistically significant.

## Results

There were 1339 participants in the case group (668) and the control group (671) enrolled in this study. The average ages of the case and control groups were 60.18 ± 9.91 years and 59.87 ± 9.30 years (Table 1). The number of males and females in the case group was 460 (68.9%) and 208 (31.1%), and in the control group were 457 (68.1%) and 214 (31.9%), respectively. The number of smoking and non-smoking participants in the case group was 351 (52.5%) and 317 (47.5%), and in the control group were 357 (53.2%) and 314 (46.8%), respectively. The number of drinking and non-drinking participants in the case group was 351 (52.5%) and 317 (47.5%), and in the control group were 361 (53.8%) and 310 (46.2%), respectively. The basic information about the participants can be found in Table 1.

**Table 1:** Characteristics of patients with LC and healthy individuals

**Characteristics**		**Cases**	**Control**	***p***
		***n* = 668**	***n* = 671**	
Age (years)	Mean ± SD	60.18 ± 9.91	59.87 ± 9.30	0.058^a^
	≤ 60	318 (47.6%)	302 (45.0%)	
	**>** 60	350 (52.4%)	369 (55.0%)	
Gender	Male	460 (68.9%)	457 (68.1%)	0.766^b^
	Female	208 (31.1%)	214 (31.9%)	
Smoking status	Yes	351 (52.5%)	357 (53.2%)	0.809^b^
	No	317 (47.5%)	314 (46.8%)	
Drinking status	Yes	351 (52.5%)	361 (53.8%)	0.645^b^
	No	317 (47.5%)	310 (46.2%)	
Tumor staging	III/IV	397 (59.0%)	-	-
	I/II	270 (40.0%)	-	-
Metastasis	Yes	380 (56.9%)	-	-
	No	64 (9.6%)	-	-
Type of cancer	LUSC	215 (32.2%)	-	-
	LUAD	325 (48.7%)	-	-

*LC* Lung cancer, *LUSC* Lung squamous cell carcinoma, *LUAD* Lung adenocarcinoma

^a^represents the *p* value calculated by the t-test

^b^represents the *p* value calculated by the chi-square test

‘-’represents the absence of these characteristics in the control group

### Genotyping and information about candidate SNPs

Three *CYP4B1* candidate genetic loci (rs2297810 A/G, rs4646491 T/C, and rs2297809 T/C) have been successfully genotyped. HaploReg showed that the three candidate SNPs were all missense variants in *CYP4B1*. The minor allele frequencies of candidate SNPs in the AFR (African), EUR (European), CHB, and CHS (Han Chinese in the Beijing/Shanghai population) have been obtained through e!Ensembl genome browser. The results showed that the MAF of candidate SNPs is different in populations with different genetic backgrounds (Table 2). Candidate genetic loci were in accordance with Hardy-Weinberg equilibrium (HWE *p* > 5%). We have used HaploReg online software to predict the potential functions of genetic loci and found that missense variants in *CYP4B1* may be regulated by a variety of factors (Table 2).

**Table 2:** The basic information and HWE about the candidate SNPs of *CYP4B1*

**SNP ID**	**Function**	**Amino acid**	**Chr: Position**	**Alleles** **(A/B)**	**MAF**		**MAF in different populations**				**HWE** **(*P* Value)**	**Haploreg 4.1**
					**Cases**	**Controls**	**CHB**	**CHS**	**AFR**	**EUR**		
rs2297810	missense	M (Met) > I (Ile)	1: 46815187	A/G	0.242	0.230	0.335	0.333	0.461	0.131	0.103	SiPhy cons; Enhancer histone marks; Motifs changed; GRASP eQTL hits; Selected eQTL hits
rs4646491	missense	R (Arg) > S (Ser)	1: 46815212	T/C	0.232	0.230	0.330	0.305	0.039	0.130	0.103	SiPhy cons; Enhancer histone marks; Motifs changed; GRASP eQTL hits; Selected eQTL hits
rs2297809	missense	R (Arg) > C (Cys)	1: 46817100	T/C	0.231	0.229	0.330	0.300	0.130	0.126	0.100	SiPhy cons; Motifs changed; Selected eQTL hits

*Met* Methionine, *Ile* Isoleucine, *Arg* Arginine, *Ser* Serine, *Cys* Cystine, *A* minor allele, *B* wild-type allele, *HWE* Hardy–Weinberg equilibrium, *SNP* Single nucleotide polymorphisms, *MAF* Minor allele frequency, *CHB* Han Chinese in Beijing, China, *CHS* Han Chinese in Shanghai, China, *EUR* European, *AFR* African

*P* > 0.05 indicates that the genotypes were in Hard-weinberg Equilibrium

### Association analysis between CYP4B1 SNPs and lung cancer susceptibility (overall analysis)

#### Overall analysis

The association analysis showed that three missense variants in *CYP4B1* were all associated with LC susceptibility (Table 3). Specifically, *CYP4B1*-rs2297810 have a significant association with the susceptibility of LC (codominant: GA Vs. GG, OR (95% CI) = 1.35 (1.08-1.69), *p* = 0.010; overdominant: GA Vs GG-AA, OR (95% CI) = 1.39 (1.11-1.73), *p* = 0.004). *CYP4B1*-rs4646491 is significantly associated with the susceptibility of LC (overdominant: CT Vs. CC-TT, OR (95% CI) = 1.30 (1.04-1.62), *p* = 0.023). *CYP4B1*-rs2297809 also had a significant association with the susceptibility of LC (codominant: TC Vs. CC, OR (95% CI) = 1.26 (1.01-1.59), *p* = 0.046; overdominant: CT Vs. CC-TT, OR (95% CI) = 1.31 (1.04-1.63), *p* = 0.020).

**Table 3:** Missense variants in *CYP4B1* associated with susceptibility of LC

**SNP ID**	**Model**	**Genotype**	**control**	**case**	**Overall analysis**	
					**OR (95% CI)**	***p*-value**
rs2297810	Allele	G	1035 (77.01%)	1011 (75.79%)	1	
		A	309 (22.99%)	323 (24.21%)	1.07 (0.9-1.28)	0.457
	Codominant	GG	405 (60.4%)	370 (55.4%)	1	
		AA	43 (6.4%)	25 (3.7%)	0.62 (0.37-1.05)	0.074
		GA	223 (33.2%)	273 (40.9%)	1.35 (1.08-1.69)	**0.010**
	Dominant	GG	405 (60.4%)	370 (55.4%)	1	
		GA-AA	266 (39.6%)	298 (44.6%)	1.22 (0.98-1.52)	0.071
	Overdominant	GG-AA	448 (66.8%)	395 (59.1%)	1	
		GA	223 (33.2%)	273 (40.9%)	1.39 (1.11-1.73)	**0.004**
	Log-additive	---	---	---	1.06 (0.89-1.27)	0.510
rs4646491	Allele	C	1035 (77.01%)	1024 (76.76%)	1	
		TT	309 (22.99%)	310 (23.24%)	1.01 (0.85-1.21)	0.879
	Codominant	CC	405 (60.4%)	382 (57.2%)	1	
		TT	43 (6.4%)	24 (3.6%)	0.98 (0.34-0.98)	0.056
		CT	223 (33.2%)	262 (39.2%)	1.26 (1.00-1.58)	0.051
	Dominant	CC	405 (60.4%)	382 (57.2%)	1	
		CT-TT	266 (39.6%)	286 (42.8%)	1.14 (0.91-1.41)	0.250
	Overdominant	CC-TT	448 (66.8%)	406 (60.8%)	1	
		CT	223 (33.2%)	262 (39.2%)	1.30 (1.04-1.62)	**0.023**
	Log-additive	---	---	---	1.01 (0.84-1.21)	0.950
rs2297809	Allele	C	1033 (77.09%)	1023 (76.92%)	1	
		T	307 (22.91%)	307 (23.08%)	1.01 (0.84-1.21)	0.916
	Codominant	CC	405 (60.5%)	382 (57.4%)	1	
		TT	43 (6.4%)	23 (3.5%)	1.05 (0.83-1.14)	0.059
		CT	221 (33%)	261 (39.2%)	1.26 (1.01-1.59)	**0.046**
	Dominant	CC	405 (60.5%)	382 (57.4%)	1	
		TC-TT	264 (39.5%)	284 (42.6%)	1.14 (0.91-1.41)	0.250
	Overdominant	CC-TT	448 (67%)	405 (60.8%)	1	
		CT	221 (33%)	261 (39.2%)	1.31 (1.04-1.63)	**0.020**
	Log-additive	---	---	---	1.00 (0.83-1.20)	0.990

*LC* Lung cancer, *LUSC* Lung squamous cell carcinoma, *LUAC* Lung adenocarcinoma, *SNP* Single nucleotide polymorphisms, *OR* Odds ratio, *CI* Confidence interval

“-” indicates Log-additive model

‘*p-*value < 0.05’ and bold text represent statistical significance

### Association analysis between CYP4B1 SNPs and LC risk (stratified analysis)

**Fig. 2:**
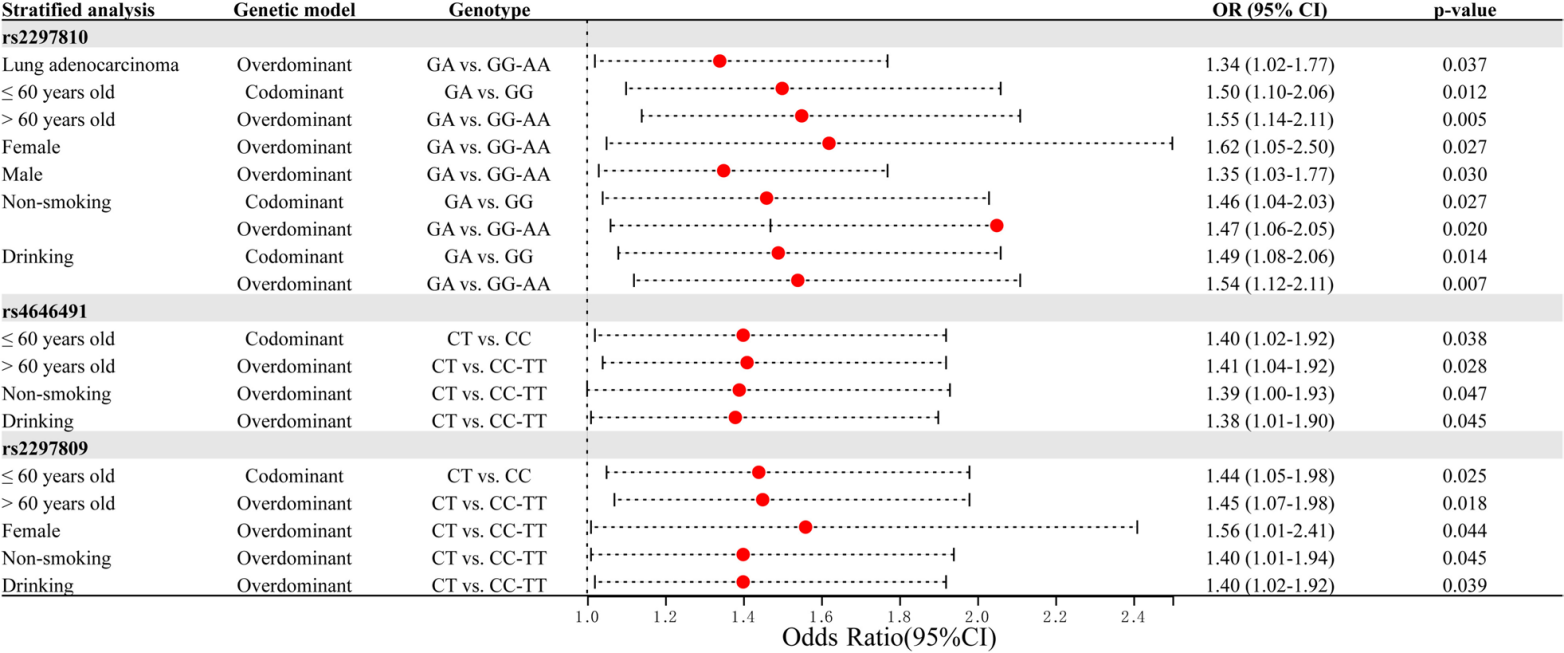
Forest map: all positive results found in stratified analysis. The main information included in the figure includes stratification information, genetic model, genotype, OR (Odds Ratio), 95%CI (95% Confidence interval), and *p*-value

The positive results in the stratified analysis of this study are all shown in Fig. 2.

#### Lung adenocarcinoma

The association analysis (Fig. 2) showed that there was only *CYP4B1*-rs2297810 significantly associated with the susceptibility of LUAD under the overdominant genetic model.

#### Lung squamous cell carcinoma

The results (Supplemental Table 2) showed that there was no genetic variant significantly associated with the susceptibility of LUSC.

#### Age (≤ 60 years old)

The analysis showed (Fig. 2) that genotype ‘GA’ of *CYP4B1*-rs2297810, ‘CT’ of -rs4646491, and ‘CT’ of -rs2297809 are all associated with LC susceptibility among participants ≤ 60 years old.

#### Age (> 60 years old)

The analysis showed (Fig. 2) that three missense variants in *CYP4B1* were all potentially associated with LC susceptibility among participants > 60 years old. Specifically, under the overdominant genetic model, *CYP4B1*-rs2297810, *CYP4B1*-rs4646491, and *CYP4B1*-rs2297809 all have a significant association with the susceptibility of LC.

#### Gender (Female)

Among female participants (Fig. 2), *CYP4B1*-rs2297810 is significantly associated with the susceptibility of LC under the overdominant genetic model Similarly, *CYP4B1*-rs2297809 is significantly associated with the susceptibility of LC under the overdominant genetic model.

#### Gender (Male)

We have found evidence (Fig. 2) that missense variant rs2297810 in *CYP4B1* were potentially associated with LC susceptibility among male participants.

#### Smoking (No)

Among non-smoking participants (Fig. 2), *CYP4B1*-rs2297810 had a significant association with the susceptibility of LC under codominant and overdominant genetic models. *CYP4B1*-rs4646491 is significantly associated with the susceptibility of LC under the overdominant genetic model. *CYP4B1*-rs2297809 also has a significant association with the susceptibility of LC under the overdominant genetic model.

#### Drinking (Yes)

We have found evidence that three missense variants in *CYP4B1* were potentially associated with LC susceptibility among drinking participants (Fig. 2). Genotype ‘GA’ of *CYP4B1*-rs2297810 had a significant association with the susceptibility of LC. *CYP4B1*-rs4646491 is also significantly associated with the susceptibility to LC under the overdominant genetic model. *CYP4B1*-rs2297809 had a potential association with the susceptibility of LC.

In addition, we have performed stratified analysis after dividing LC patients according to tumor stage and cancer metastasis. The analysis showed that there were no SNPs associated with LC susceptibility in the above two stratified analyses (Supplemental Table 2).

### FPRP analysis

FPRP analysis showed that all positive results are noteworthy findings at the prior probability level of 0.25 and FPRP threshold of 0.2 (Supplemental Table 3). The statistical power of positive results in this study ranged from 82.9% to 100%. Especially in the overall analysis, the statistical power is above 99.9%.

### MDR analysis

**Fig. 3:**
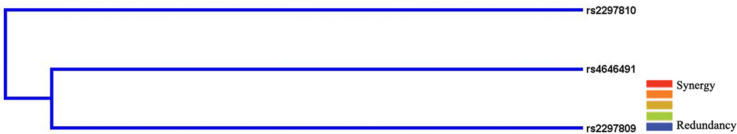
Multifactor dimensionality reduction (MDR) analysis of interaction between the candidate SNPs of *CYP4B1* (rs2297810, rs4646491, and rs2297809). The color represents the degree of redundancy or synergy between SNP-SNP; the closer the color is to red, the more synergy, and the closer to blue, the more redundancy

As is shown in Fig. 3, the dendrogram has described the interaction between the three candidate SNPs. The color of the lines in the dendrogram represents the level of redundancy or synergy. The interaction between the three candidate SNPs is redundant. The MDR results showed that a single loci model composed of rs2297810 was chosen as the best model for predicting LC risk (*p* = 0.0046), with the best test accuracy of 0.537 and a perfect CVC = 10/10 (Table 4).

**Table 4:** *CYP4B1* SNP–SNP interaction models analyzed by the MDR method

**Model**	**Training Bal. Acc**	**Testing Bal. Acc**	**OR (95% CI)**	***p* value**	**CVC**
rs2297810	0.537	0.537	1.38 (1.10 - 1.72)	**0.0046**	10/10
rs2297810,rs2297809	0.538	0.534	1.39 (1.11 - 1.73)	**0.0039**	7/10
rs2297810,rs4646491,rs2297809	0.538	0.532	1.39 (1.11 -1.73)	**0.0039**	10/10

*MDR* Multifactor dimensionality reduction, *Bal. Acc.* Balanced accuracy, *CVC* Cross-validation consistency, *OR* Odds ratio, *95% CI* 95% confidence interval.

*p* values were calculated using χ^2^ tests; ‘*p-*value < 0.05’ and bold text represent statistical significance.

### The association between genotype and gene expression level

**Fig. 4:**
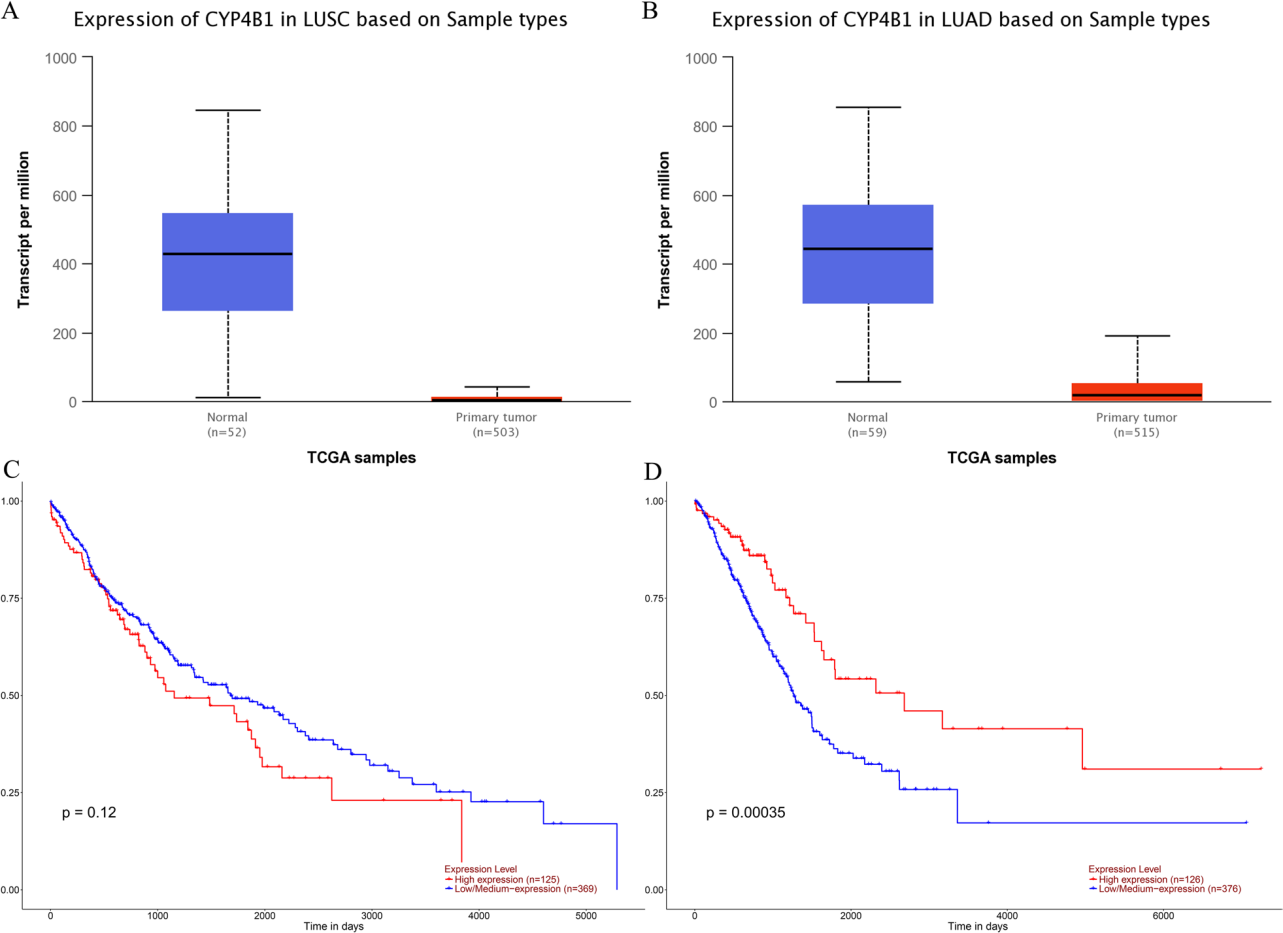
Prediction of the expression and prognosis of *CYP4B1* gene in LUSC and LUAD. **A** Expression of *CYP4B1* in LUSC and normal tissues (*p* = 1.62E-12). **B** Expression of CYP4B1 in LUAD and normal tissues (*p* = 1.62E-12). **C** Effect of *CYP4B1* gene expression on prognosis of LUSC (*p* = 0.12). **D** Effect of *CYP4B1* gene expression on prognosis of LUAD (*p* = 0.00035)

**Fig. 5:**
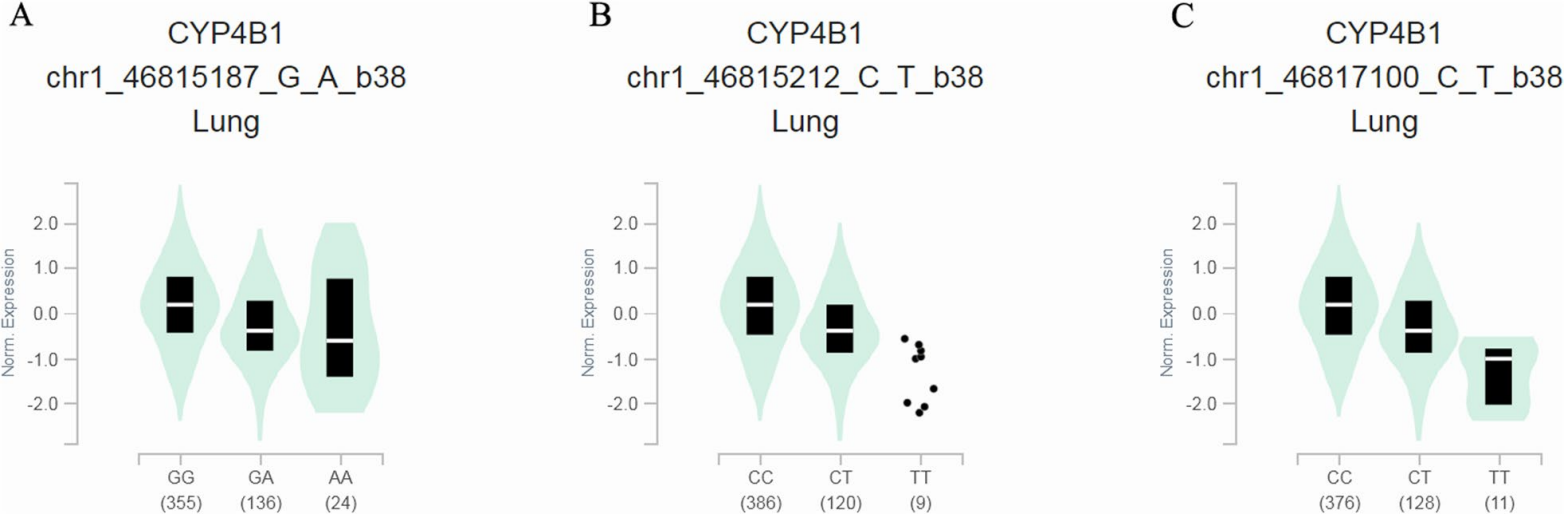
Expression of *CYP4B1* under rs2297810 genotype in the lung tissues; (**B**) Expression of *CYP4B1* under rs4646491 genotype in the lung tissues; (**C**) Expression of *CYP4B1* under rs2297809 genotype in the lung tissues.

As shown in Fig. 4A and B, *CYP4B1* expression levels in LUSC were significantly lower than in normal lung tissues (*p* = 1.62E-12). Similarly, *CYP4B1* expression levels in LUAD were significantly lower than in normal lung tissues (*p* = 1.62E-12). In addition, we found that *CYP4B1* expression level was not associated with the prognosis of LUSC (*p* = 0.12; Fig. 4C), but*CYP4B1* expression level was significantly associated with the prognosis of LUAD (*p* = 0.00035; Fig. 4D). Furthermore, the GTEx prediction analysis showed (Fig. 5) that different genotypes of each candidate genetic loci are significantly associated with the expression level of *CYP4B1*.

## Discussion

We conducted a study on the association between three missense variants in *CYP4B1* and LC susceptibility in 1339 participants. Combined with correlation analysis and FPRP results, three candidate missense variants in *CYP4B1* (rs2297810, rs4646491, and rs2297809) were found to be associated with LC risk. The genotype GA of *CYP4B1*-rs2297810 was significantly associated with an increased risk of lung cancer in both overall and stratified analyses. Similarly, genotype CT of *CYP4B1*-rs4646491 and genotype CT of *CYP4B1*-rs2297809 are also associated with an increased risk of lung cancer. These indicate that *CYP4B1*-rsS2297810, -rs4646491, and -rs2297809 are potential genetic risk factors for lung cancer. To our knowledge, this study is the first to report that *CYP4B1* genetic polymorphisms are associated with lung cancer susceptibility in the Chinese Han population.

The analysis showed that the presence of these three missense variant heterozygous genotypes will increase the risk of lung cancer whether participants were younger or older than 60 years. Genotype GA of *CYP4B1*-rs2297810 is a risk factor for both female and male lung cancer. *CYP4B1*-rs2297809 is associated with an increased risk of female lung cancer. Although no significant results of CYP4B1-rs2297809 associated with lung cancer were found in the male population, the overall trend was that the presence of the CT genotype of *CYP4B1*-rs2297809 will also increase the risk of male lung cancer (OR > 1). We also found that the three missense variants in *CYP4B1* were significantly associated with an increased risk of lung cancer in non-smokers, while no positive results were found in smokers. Age, smoking, and gender differences have previously been reported as risk factors for lung cancer [34–36]. Czerwinski M, et al have found that *CYP4B1* is not induced by compounds present in cigarette smoke in lung cancer patients [37]. Combined with previous studies and the results of our study, we speculated that the three missense variants in *CYP4B1* are risk factors for lung cancer in the Chinese Han population, and the above genetic risk factors related to *CYP4B1* may not be affected by these potential risk factors.

In recent years, research on *CYP4B1* in cancer has attracted special attention, which may be due to its different expression in patients with various cancers, including lung cancer, compared with normal individuals [23, 37, 38]. *CYP4B1* was significantly downregulated in lung cancer patients, which was further confirmed by UALCAN online database analysis in this study. In addition, through the database, we also found that low expression of *CYP4B1* was significantly associated with the prognosis of LUAD patients. Thus, the expression level of *CYP4B1* is closely related to lung cancer, but its regulation mechanism in lung cancer is still not clear. Searching in the genotype-tissue expression database showed that *CYP4B1* expression levels were different in lung tissues under different genotypes of these missense variants. And we found that these variants in *CYP4B1* can cause changes in amino acid sequence when we used the dbSNP database to search the information related to candidate genetic variants. Changes in amino acid sequence can cause changes in protein structure, which is directly related to its function [39]. In addition, *CYP4* is involved in fatty acid metabolism, and the reprogramming of fatty acid metabolism is very important for the occurrence of lung cancer [25]. Combined with previous studies and the results of our study, we speculate that these missense variants may cause changes in the amino acid sequence, which may cause changes in the protein structure of *CYP4B1*, thereby affecting the gene expression level, thereby affecting the fatty acid metabolism process, and ultimately affecting the LC risk. However, the above is only speculation, and further mechanism research is necessary to explore how these three candidate missense variants affect the susceptibility to LC by affecting the expression of *CYP4B1* among the Chinese Han population. In any case, this study has laid a reliable theoretical foundation for the mechanism of *CYP4B1* in the development of lung cancer. At the same time, it has provided new ideas for risk assessment and clinical individualized prevention and treatment of lung cancer among Chinese Han.

However, we must face the fact that this study has certain limitations. First, it is necessary to conduct a large sample size or confirmatory study in people with different genetic backgrounds, which will help ensure the reproducibility and reliability of the results of this study. Secondly, further design of functional validation tests will help to accurately understand the mechanism of three *CYP4B1* gene polymorphisms in the occurrence and development of lung cancer. The above studies will further help us understand the potential molecular mechanism of the three *CYP4B1* genetic loci in LC risk, which in turn will help to further understand the pathogenesis of LC. We believe this will be a very interesting research direction. In any case, this study is the first to explore the association of *CYP4B1* gene polymorphism with lung cancer susceptibility in the Chinese Han population and has achieved positive results.

## Conclusion

In summary, this study showed that missense variants in *CYP4B1* (rs2297810, rs4646491, and rs2297809) were associated with LC susceptibility. Especially when these genetic variants are under heterozygous genotypes, the risk of lung cancer may be increased in Chinese Han population. This study provides a new research idea and lays a reliable theoretical foundation for the early diagnosis and individualized treatment of lung cancer in Chinese Han population.

### Supplementary Information

**Additional file 1.**

## Data Availability

The datasets used and analyzed in the current study are available from the corresponding author on reasonable request.
