# Supplementary material for: Missense variants in CYP4B1 associated with increased risk of lung cancer among Chinese Han population
Source: World J Surg Oncol. 2023 Nov 11;21:352. doi: 10.1186/s12957-023-03223-2 (PMC10638751; doi:10.1186/s12957-023-03223-2)
Supplement: Supplementary file 1 — Additional file 1. [file 12957_2023_3223_MOESM1_ESM.docx]

**Supplemental table 1** The primers for *CYP4B1*gene amplification and sequencing.

| **Gene** | **SNP** | **1st-PCR primer** | **2nd-PCR primer** | **UEP-DIR** | **UEP-SEQ** |
| --- | --- | --- | --- | --- | --- |
| *CYP4B1* | rs2297810 | ACGTTGGATGTTTGAAGGCCATGACACCAC | ACGTTGGATGCGGACCTCCTCTCTACAAC | R | CTCAGGGTACAGGGC |
|  | rs4646491 | ACGTTGGATGTCCTGGTCCCCTAGGATCTC | ACGTTGGATGGTGGTATCTCCTGGTTTCTC | F | CCTGAGCACCAGCAT |
|  | rs2297809 | ACGTTGGATGGGGATGATCTGGGCAAAATG | ACGTTGGATGAAGGTGACAGGCTTGCTGAG | R | CACAGGTGGGTAGAGGC |

SNP, single nucleotide polymorphism; UEP-DIR, Unique extension primer-direction; UEP-SEQ, Unique extension primer-sequencing.

**Supplemental Table 2** Association analysis between missense variants in *CYP4B1* and susceptibility to LC in the subgroup analysis (Tumor staging and cancer metastasis).

| **SNP ID** | **Model** | **Genotype** | **LUSC** | | | |  | **Tumor staging (III/IV Vs. I/II )** | | | |  | **Cancer metastasis (Yes Vs. No)** | | | |
| --- | --- | --- | --- | --- | --- | --- | --- | --- | --- | --- | --- | --- | --- | --- | --- | --- |
|  |  |  | **control** | **case** | **OR (95% CI)** | ***p*-value** |  | **control** | **case** | **OR (95% CI)** | ***p*-value** |  | **control** | **case** | **OR (95% CI)** | ***p*-value** |
| rs2297810 | Allele | G | 1035 (77.01%) | 327 (76.05%) | 1 |  |  | 422 (78.15%) | 590 (74.31%) | 1 |  |  | 107 (83.59%) | 570 (75.00%) | 1 |  |
|  |  | A | 309 (22.99%) | 103 (23.95%) | 1.05 (0.82-1.36) | 0.692 |  | 118 (21.85%) | 204 (25.69%) | 1.24 (0.95-1.6) | 0.108 |  | 21 (16.41%) | 190 (25.00%) | 1.7 (1.04-2.19) | 0.051 |
|  | Codominant | GG | 405 (60.4%) | 123 (57.2%) | 1 |  |  | 139 (51.5%) | 230 (57.9%) | 1 |  |  | 38 (59.4%) | 219 (57.6%) | 1 |  |
|  |  | AA | 43 (6.4%) | 85 (39.5%) | 0.69 (0.32-1.50) | 0.350 |  | 119 (44.1%) | 154 (38.8%) | 1.42 (0.6-3.33) | 0.421 |  | 26 (40.6%) | 144 (37.9%) | / | / |
|  |  | GA | 223 (33.2%) | 7 (3.3%) | 1.28 (0.91-1.78) | 0.152 |  | 12 (4.4%) | 13 (3.3%) | 1.3 (0.94-1.79) | 0.114 |  | 0 (0%) | 17 (4.5%) | 1.51 (0.86-2.67) | 0.154 |
|  | Dominant | GG | 405 (60.4%) | 123 (57.2%) | 1 |  |  | 139 (51.5%) | 230 (57.9%) | 1 |  |  | 38 (59.4%) | 219 (57.6%) | 1 |  |
|  |  | GA-AA | 266 (39.6%) | 92 (42.8%) | 1.12 (0.81-1.53) | 0.500 |  | 131 (48.5%) | 167 (42.1%) | 0.77 (0.56-1.05) | 0.100 |  | 26 (40.6%) | 161 (42.4%) | 1.03 (0.60-1.78) | 0.920 |
|  | Overdominant | GG-AA | 448 (66.8%) | 130 (60.5%) | 1 |  |  | 151 (55.9%) | 243 (61.2%) | 1 |  |  | 38 (59.4%) | 236 (62.1%) | 1 |  |
|  |  | GA | 223 (33.2%) | 85 (39.5%) | 1.27 (0.92-1.76) | 0.150 |  | 119 (44.1%) | 154 (38.8%) | 0.80 (0.59-1.10) | 0.180 |  | 26 (40.6%) | 144 (37.9%) | 0.87 (0.50-1.50) | 0.620 |
|  | Log-additive | --- | --- | --- | 0.99 (0.76-1.29) | 0.960 |  | --- | --- | 0.79 (0.60-1.04) | 0.092 |  | --- | --- | 1.17 (0.71-1.90) | 0.540 |
| rs4646491 | Allele | C | 1035 (77.01%) | 328 | 1 |  |  | 426 (78.89%) | 599 (75.44%) | 1 |  |  | 107 (83.59%) | 581 (76.45%) | 1 |  |
|  |  | T | 309 (22.99%) | 102 | 1.04 (0.80-1.34) | 0.766 |  | 114 (21.11%) | 195 (24.56%) | 1.22 (0.94-1.58) | 0.143 |  | 21 (16.41%) | 179 (23.585%) | 1.57 (0.96-2.58) | 0.073 |
|  | Codominant | CC | 405 (60.4%) | 129 (60%) | 1 |  |  | 142 (52.6%) | 239 (60.2%) | 1 |  |  | 41 (64.1%) | 222 (58.4%) | 1 |  |
|  |  | TT | 43 (6.4%) | 79 (36.7%) | 0.69 (0.32-1.48) | 0.340 |  | 117 (43.3%) | 145 (36.5%) | 1.28 (0.54-3.04) | 0.576 |  | 23 (35.9%) | 143 (37.6%) | / | / |
|  |  | CT | 223 (33.2%) | 7 (3.3%) | 1.24 (0.89-1.73) | 0.200 |  | 11 (4.1%) | 13 (3.3%) | 1.28 (0.92-1.77) | 0.139 |  | 0 (0%) | 15 (4%) | 1.35 (0.76-2.39) | 0.302 |
|  | Dominant | CC | 405 (60.4%) | 129 (60%) | 1 |  |  | 142 (52.6%) | 239 (60.2%) | 1 |  |  | 41 (64.1%) | 222 (58.4%) | 1 |  |
|  |  | CT-TT | 266 (39.6%) | 86 (40%) | 0.99 (0.72-1.37) | 0.960 |  | 128 (47.4%) | 158 (39.8%) | 0.73 (0.53-1.00) | 0.048 |  | 23 (35.9%) | 158 (41.6%) | 1.21 (0.69-2.11) | 0.500 |
|  | Overdominant | CC-TT | 448 (66.8%) | 136 (63.3%) | 1 |  |  | 153 (56.7%) | 252 (63.5%) | 1 |  |  | 41 (64.1%) | 237 (62.4%) | 1 |  |
|  |  | CT | 223 (33.2%) | 79 (36.7%) | 1.13 (0.82-1.57) | 0.460 |  | 117 (43.3%) | 145 (36.5%) | 0.75 (0.54-1.03) | 0.071 |  | 23 (35.9%) | 143 (37.6%) | 1.04 (0.59-1.81) | 0.900 |
|  | Log-additive | --- | --- | --- | 0.91 (0.70-1.19) | 0.500 |  | --- | --- | 0.77 (0.58-1.01) | 0.058 |  | --- | --- | 1.32 (0.79-2.19) | 0.280 |
| rs2297809 | Allele | C | 1033 (77.09%) | 327 (76.40%) | 1 |  |  | 425 (79.00%) | 599 (75.63%) | 1 |  |  | 107 (83.59%) | 579 (76.39%) | 1 |  |
|  |  | T | 307 (22.91%) | 101 (23.60%) | 1.04 (0.8-1.34) | 0.780 |  | 113 (21.00%) | 193 (24.37%) | 1.21 (0.93-1.58) | 0.152 |  | 21 (16.41%) | 179 (23.61%) | 1.58 (0.96-2.59) | 0.071 |
|  | Codominant | CC | 405 (60.5%) | 128 (59.8%) | 1 |  |  | 142 (52.8%) | 239 (60.4%) | 1 |  |  | 41 (64.1%) | 221 (58.5%) | 1 |  |
|  |  | TT | 43 (6.4%) | 80 (37.4%) | 0.6 (0.27-1.35) | 0.217 |  | 116 (43.1%) | 145 (36.6%) | 1.2 (0.5-2.88) | 0.682 |  | 23 (35.9%) | 142 (37.6%) | / | / |
|  |  | TC | 221 (33%) | 6 (2.8%) | 1.28 (0.92-1.78) | 0.147 |  | 11 (4.1%) | 12 (3%) | 1.29 (0.93-1.79) | 0.121 |  | 0 (0%) | 15 (4%) | 1.36 (0.77-2.4) | 0.289 |
|  | Dominant | CC | 405 (60.5%) | 128 (59.8%) | 1 |  |  | 142 (52.8%) | 239 (60.4%) | 1 |  |  | 41 (64.1%) | 221 (58.5%) | 1 |  |
|  |  | TC-TT | 264 (39.5%) | 86 (40.2%) | 1.00 (0.73-1.38) | 0.990 |  | 127 (47.2%) | 157 (39.6%) | 0.73 (0.53-1.00) | 0.050 |  | 23 (35.9%) | 157 (41.5%) | 1.21 (0.69-2.10) | 0.510 |
|  | Overdominant | CC-TT | 448 (67%) | 134 (62.6%) | 1 |  |  | 153 (56.9%) | 251 (63.4%) | 1 |  |  | 41 (64.1%) | 236 (62.4%) | 1 |  |
|  |  | TC | 221 (33%) | 80 (37.4%) | 1.17 (0.84-1.62) | 0.360 |  | 116 (43.1%) | 145 (36.6%) | 0.76 (0.55-1.04) | 0.088 |  | 23 (35.9%) | 142 (37.6%) | 1.03 (0.59-1.81) | 0.910 |
|  | Log-additive | --- | --- | --- | 0.91 (0.69-1.19) | 0.480 |  | --- | --- | 0.76 (0.58-1.00) | 0.050 |  | --- | --- | 1.32 (0.79-2.19) | 0.280 |

SNP: Single nucleotide polymorphisms; LUSC, lung squamous cell carcinoma; OR, Odds ratio; CI, Confidence interval.

“-” indicates Log-additive model; ‘/’ indicates that the data is missing.

‘*p-*value < 0.05’ and bold text represent statistical significance.

**Supplemental table 3** The FPRP and statistical power values of all the positive results in this study.

| **SNP ID** | **Model** | **Genotype** | **OR (95%CI)** | ***p*** | **Statistical Power ^a^ (%)** | **Prior probability** | | |
| --- | --- | --- | --- | --- | --- | --- | --- | --- |
|  |  |  |  |  |  | **0.25** | **0.1** | **0.01** |
| **Overall analysis** |  |  |  |  |  |  |  |  |
| rs2297810 | Codominant | GA | 1.35 (1.08-1.69) | **0.010** | 100.0% | 0.026* | 0.074* | 0.466 |
|  | Overdominant | GA | 1.39 (1.11-1.73) | **0.004** | 99.9% | 0.009* | 0.028* | 0.240 |
| rs4646491 | Overdominant | CT | 1.30 (1.04-1.62) | **0.023** | 100.0% | 0.055* | 0.149* | 0.658 |
| rs2297809 | Codominant | TC | 1.26 (1.01-1.59) | **0.046** | 100.0% | 0.134* | 0.317 | 0.836 |
|  | Overdominant | TC | 1.31 (1.04-1.63) | **0.020** | 100.0% | 0.044* | 0.122* | 0.605 |
| **Female** |  |  |  |  |  |  |  |  |
| rs2297810 | Overdominant | GA | 1.62 (1.05-2.50) | **0.027** | 82.9% | 0.096* | 0.241 | 0.778 |
| rs2297809 | Overdominant | TC | 1.56 (1.01-2.41) | **0.044** | 86.9% | 0.135* | 0.318 | 0.837 |
| **Male** |  |  |  |  |  |  |  |  |
| rs2297810 | Overdominant | GA | 1.35 (1.03-1.77) | **0.030** | 99.8% | 0.082* | 0.212 | 0.748 |
| **≤ 60 years old** |  |  |  |  |  |  |  |  |
| rs2297810 | Codominant | GA | 1.50 (1.10-2.06) | **0.012** | 96.2% | 0.037* | 0.103* | 0.557 |
| rs4646491 | Codominant | CT | 1.40 (1.02-1.92) | **0.038** | 98.7% | 0.101* | 0.251 | 0.787 |
| rs2297809 | Codominant | TC | 1.44 (1.05-1.98) | **0.025** | 97.8% | 0.071* | 0.186* | 0.715 |
| **> 60 years old** |  |  |  |  |  |  |  |  |
| rs2297810 | Overdominant | GA | 1.55 (1.14-2.11) | **0.005** | 94.7% | 0.017* | 0.048* | 0.359 |
| rs4646491 | Overdominant | CT | 1.41 (1.04-1.92) | **0.028** | 98.7% | 0.081* | 0.210 | 0.745 |
| rs2297809 | Overdominant | TC | 1.45 (1.07-1.98) | **0.018** | 97.8% | 0.056* | 0.151* | 0.663 |
| **Smoking (No)** |  |  |  |  |  |  |  |  |
| rs2297810 | Codominant | GA | 1.46 (1.04-2.03) | **0.027** | 96.9% | 0.070* | 0.185* | 0.714 |
|  | Overdominant | GA | 1.47 (1.06-2.05) | **0.020** | 96.5% | 0.067* | 0.178* | 0.704 |
| rs4646491 | Overdominant | CT | 1.39 (1.00-1.93) | **0.047** | 98.5% | 0.130* | 0.310 | 0.832 |
| rs2297809 | Overdominant | TC | 1.40 (1.01-1.94) | **0.045** | 98.4% | 0.116* | 0.283 | 0.813 |
| **Drinking (Yes)** |  |  |  |  |  |  |  |  |
| rs2297810 | Codominant | GA | 1.49 (1.08-2.06) | **0.014** | 96.3% | 0.047* | 0.129* | 0.619 |
|  | Overdominant | GA | 1.54 (1.12-2.11) | **0.007** | 94.8% | 0.022* | 0.064* | 0.429 |
| rs4646491 | Overdominant | CT | 1.38 (1.01-1.90) | **0.045** | 98.9% | 0.128* | 0.306 | 0.829 |
| rs2297809 | Overdominant | TC | 1.40 (1.02-1.92) | **0.039** | 98.7% | 0.101* | 0.251 | 0.787 |
| **Lung adenocarcinoma Vs. controls** |  |  |  |  |  |  |  |  |
| rs2297810 | Overdominant | GA | 1.34 (1.02-1.77) | **0.037** | 99.8% | 0.106* | 0.262 | 0.796 |

FPRP: false-positive report probability.

^a^ Statistical power ^a^ was calculated using the number of observations in the subgroup and the OR and p values in this table.

^b^ The level of false-positive report probability threshold was set at 0.2, and ‘*’ indicate noteworthy findings.
